# Burning the candle at both ends: Intraretinal signaling of intrinsically photosensitive retinal ganglion cells

**DOI:** 10.3389/fncel.2022.1095787

**Published:** 2023-01-06

**Authors:** Sushmitha Raja, Nina Milosavljevic, Annette E. Allen, Morven A. Cameron

**Affiliations:** ^1^School of Medicine, Western Sydney University, Sydney, NSW, Australia; ^2^Division of Neuroscience, Faculty of Biology, Medicine and Health, The University of Manchester, Manchester, United Kingdom

**Keywords:** melanopsin, intrinsically photosensitive retinal ganglion cells (ipRGCs), retina, intraretinal, retinal processing

## Abstract

Intrinsically photosensitive retinal ganglion cells (ipRGCs) are photoreceptors located in the ganglion cell layer. They project to brain regions involved in predominately non-image-forming functions including entrainment of circadian rhythms, control of the pupil light reflex, and modulation of mood and behavior. In addition to possessing intrinsic photosensitivity via the photopigment melanopsin, these cells receive inputs originating in rods and cones. While most research in the last two decades has focused on the downstream influence of ipRGC signaling, recent studies have shown that ipRGCs also act retrogradely within the retina itself as intraretinal signaling neurons. In this article, we review studies examining intraretinal and, in addition, intraocular signaling pathways of ipRGCs. Through these pathways, ipRGCs regulate inner and outer retinal circuitry through both chemical and electrical synapses, modulate the outputs of ganglion cells (both ipRGCs and non-ipRGCs), and influence arrangement of the correct retinal circuitry and vasculature during development. These data suggest that ipRGC function plays a significant role in the processing of image-forming vision at its earliest stage, positioning these photoreceptors to exert a vital role in perceptual vision. This research will have important implications for lighting design to optimize the best chromatic lighting environments for humans, both in adults and potentially even during fetal and postnatal development. Further studies into these unique ipRGC signaling pathways could also lead to a better understanding of the development of ocular dysfunctions such as myopia.

## Introduction

The discovery of a third class of photoreceptor in the retina, the intrinsically photosensitive retinal ganglion cells (ipRGCs) (Berson et al., 2002; Hattar et al., 2002) two decades ago changed the landscape of retinal biology. While most research thus far has concentrated on the downstream influence of these ipRGCs, they have also been shown to play a significant role in intra-retinal and intra-ocular signaling. This raises the intriguing possibility that ipRGCs exert a wider influence on image-forming vision by modulating the retinal output. These ganglion cell photoreceptors express the photopigment melanopsin (Provencio et al., 1998) in their soma and dendrites and form a photoreceptive net in the inner retina. ipRGCs constitute a subset of retinal ganglion cells [RGCs, approximately 3-5% of the population (Hattar et al., 2002)] and, like other RGCs, receive rod/cone light responses via synaptic connections with bipolar and amacrine cells in the inner plexiform layer (IPL). Their intrinsic light response, driven by melanopsin, has a long temporal integration (Do et al., 2009) which allows them to perform light-mediated physiological functions such as circadian photoentrainment (Freedman et al., 1999), sustained pupil light constriction (Lucas et al., 2003), and pineal melatonin suppression (Lucas et al., 1999). Acting retrogradely as intraretinal signaling neurons they have been shown to play a role in modulating the physiological processes within the retina itself through light adaptation of the retina, involvement in the retinal circadian clock, and fine-tuning of retinal circuitry. In this review, we focus on the mechanism of this local intraretinal and intraocular signaling of ipRGCs, and the processes they are driving. While the majority of these studies have been completed on laboratory rodents, we have endeavored to include work completed in primate and human retina where available.

## Intrinsically photosensitive retinal ganglion cells

Initially, it was assumed that all light signals for image and non-image-forming vision originated within rods and cones. Multiple studies over the last two decades, however, showed discrepancies in this assumption (Lucas et al., 1999). This led to the discovery of a third class of photoreceptor in the retina, the intrinsically photosensitive retinal ganglion cells (ipRGCs) (Berson et al., 2002; Hattar et al., 2002), a small subpopulation of the retinal ganglion cells expressing melanopsin pigment encoded by the *Opn4* gene (Provencio et al., 1998, 2000). By retrograde labeling using a fluorescent tracer injected in the rat suprachiasmatic nucleus (SCN), these neurons were identified and targeted for electrophysiological recordings where light responses were observed even after pharmacologically blocking rod and cone pathways (Berson et al., 2002).

Melanopsin, upon light activation, triggers a G-protein cascade causing membrane depolarization (Graham et al., 2008; Do et al., 2009; Sonoda et al., 2018). In melanopsin knockout mice, RGCs retrogradely labeled from the SCN lost their intrinsic photosensitivity but many non-image-forming responses remained intact or were only mildly impaired (Panda et al., 2002, 2003; Ruby et al., 2002; Hattar et al., 2003; Lucas et al., 2003). However, the genetic elimination of ipRGCs themselves via expression of diphtheria toxin by the melanopsin promoter led to complete loss of circadian entrainment and a severely impaired pupil light response (Goz et al., 2008; Guler et al., 2008; Hatori et al., 2008). Taken together these data indicate that ipRGCs are the principal conduits for non-image-forming vision but their intrinsic photosensitivity, *via* melanopsin, is coupled with rod and cone signals to fine-tune these non-image-forming responses. Indeed, without functional rods, cones and melanopsin, animals lack the majority of image and non-image-forming responses (Hattar et al., 2003). Further, when melanopsin was heterologously expressed in cells otherwise insensitive to light, they gained photosensitivity, indicating melanopsin’s ability to function as a *bona fide* photopigment (Melyan et al., 2005; Panda et al., 2005; Qiu et al., 2005).

While much research over the last two decades has focused on the downstream targets and influence of ipRGCs, they have also been shown to influence and modulate cells of the inner retina and perhaps even the photoreceptors. Hence, in addition to their role as output projection neurons, ipRGCs also signal retrogradely within the retina itself.

## Types of ipRGCs and their location in the retina

**FIGURE 1:**
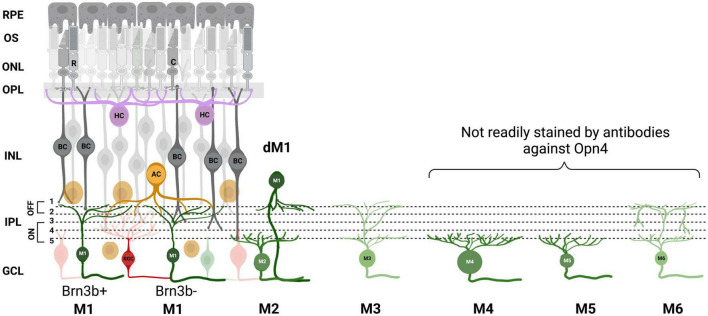
ipRGC subtypes. A, Schematic of the mouse retina with the 6 melanopsin subtypes and their stratification in the inner plexiform layer (IPL) depicted. M1 ipRGC subtype is further divided into Brn3b positive and negative, and displaced M1s (dM1). Retinal pigment epithelium (RPE), outer segment (OS), outer nuclear layer (ONL), outer plexiform layer (OPL), inner nuclear layer (INL), ganglion cell layer (GCL), rods (R), cones (C), bipolar cell (BC), amacrine cell (AC), horizontal cell (HC). Image was created with BioRender.com.

The ipRGCs initially described by Berson et al. (2002) were thought to be a morphologically homogeneous class. However, subsequent research over the years has revealed evidence of at least 6 morphologically and physiologically distinct subtypes of ipRGCs (M1-M6; Figure 1) that display different morphologies and contribute differentially to non-image-forming and image-forming behaviors. Immunolabelling of ipRGCs with melanopsin-specific antibodies has shown that they stratify in both ON and OFF sublaminae of IPL (Provencio et al., 2002). The ipRGCs originally described by Berson in 2002 belong to the M1 subtype. M1 cells stratify in the OFF sublamina of the IPL while the M2, M4, and M5 cells stratify in the ON sublamina and the M3 (Schmidt and Kofuji, 2011) and M6 (Quattrochi et al., 2019) cells have stratification in both ON and OFF sublaminae (Viney et al., 2007; Baver et al., 2008; Schmidt et al., 2008; Schmidt and Kofuji, 2009; Berson et al., 2010; Ecker et al., 2010; Quattrochi et al., 2019).

M1 subtypes have a small soma and dendrites and the strongest melanopsin expression of all types. Despite their stratification in OFF sublamina, they mostly receive synaptic input from *en passant* ON bipolar cells (Dumitrescu et al., 2009; Hoshi et al., 2009; Grünert et al., 2011). M1 ipRGCs can be further subdivided into two types based on the expression of the transcription factor Brn3b. Brn3b-positive M1 ipRGCs have been shown to provide innervation to the olivary pretectal nucleus (OPN) controlling the pupil light reflex and Brn3b-negative ipRGC provide light information to the SCN for photoentrainment (Chen et al., 2011). M1s are found in both the ganglion cell layer (GCL) and the inner nuclear layer (INL) where they are termed “displaced” M1 ipRGCs. M2 cells have a larger soma and a more highly branched dendritic tree than M1 (Schmidt and Kofuji, 2009; Berson et al., 2010; Ecker et al., 2010) but show significantly smaller amplitude intrinsic light responses and are at least 1 log unit less sensitive than M1s (Schmidt and Kofuji, 2009). They have been shown to project some fibers to the SCN, but predominately to the OPN, suggesting they play more of a role in the pupil light reflex (Baver et al., 2008). M3 cells display similar soma and dendritic size and complexity as that of M2s (Schmidt and Kofuji, 2011). M4 cells are the sustained ON-type alpha ganglion cells which have a primary role in contrast detection in image-forming vision. The presence of melanopsin in these cells acts to shape and optimize rod- and cone-driven responses (Sonoda et al., 2018). They have the largest soma of any described ipRGC and a large, highly branched dendritic arbor. M5 cells have small, highly branched bushy dendritic arbors uniformly distributed around the soma and have been shown to display color opponency (Stabio et al., 2018). M6 cells have the smallest dendritic fields with a spiny, abundantly branched dendritic arbor. M6 cells are bistratified and possess recurrent dendrites that return to stratify on the ON sublamina after traversing a short distance in the OFF sublamina. Their axons project to dLGN, suggesting they may contribute to pattern vision (Quattrochi et al., 2019). Studies show evidence that some ipRGC subtypes are conserved across species, mice to humans, with potentially parallel behavioral roles (Gamlin et al., 1995, 2007; Gamlin, 2006; Jusuf et al., 2007; Liao et al., 2016) and all subtypes of ipRGCs survive retinal degeneration in mice (Procyk et al., 2022). The authors also found one ipRGC in the *rd/rd* retina that did not fit into any of the six defined ipRGC subtypes in mice described in the literature, thanks to its very large dendritic field size (Procyk et al., 2022) but exhibited similar characteristics as “Gigantic M1” reported in the human retina (Hannibal et al., 2017). Finally, retrograde labeling from the optic nerve stained the majority of ipRGC cell bodies, but did not stain a small subset of displaced ipRGCs, suggesting they do not project axons down the optic nerve (Valiente-Soriano et al., 2014). These putative ipRGC interneurons are displaced to the INL and primarily found near the ciliary marginal zone (CMZ) suggesting that they may have a role in the intrinsic pupil light response (Schmidt et al., 2014; Semo et al., 2014).

## Synaptic connections to neighboring retinal neurons

So how might this heterogenous class of photosensitive ganglion cells be communicating to neighboring cells in the retina? The first evidence of chemical synaptic intraretinal signaling from ipRGCs was their input to dopaminergic amacrine (DA) cells of the inner nuclear layer (INL). ipRGC have been shown to provide excitatory input to these cells (Zhang et al., 2008, 2012; Liu et al., 2017) which is matched by DA to ipRGC signaling via reciprocal synapses (Viney et al., 2007; Vugler et al., 2007). DA cells are spiking amacrines and are thought to be the only source of dopamine in the retina. The release of dopamine has diverse modulatory effects on both cellular function and the organization of retinal circuitry to correctly encode visual information at different ambient light levels (Witkovsky, 2004). In the mouse retina, different functional subtypes of DA cells have been identified: those with transient light responses and those with sustained light responses (Zhang et al., 2007). The sustained DA cells (sDA) are driven by a cationic current that persists in the presence of L-AP4 (which blocks the ON- pathway from photoreceptors), and shows melanopsin response characteristics like long latencies, a marked post-stimulus persistence, and a spectral sensitivity at 478 nm. Indeed, sustained DA cell responses persisted in degenerate retinae lacking rods and cones (Zhang et al., 2008) and were absent in *Opn4^–/–^* animals (Zhang et al., 2012). This input can be blocked by DNQX (6,7-dinitroquinoxaline-2,3-dione), an AMPA/Kainate blocker (Zhang et al., 2008), and is thought to originate primarily from M1 ipRGCs via axon collaterals that synapse with TH cell processes in the OFF stratum of the IPL (Zhang et al., 2007; Joo et al., 2013; Prigge et al., 2016). These axon collaterals branch from the primary axon at varying distances from the soma (up to 800 μm) of ∼7% M1 cells. Both inner and outer stratifying collaterals were identified, suggesting these collaterals also convey information to inner stratifying ganglion or amacrine cells (Joo et al., 2013).

The discovery of this synaptic input to DA cells that originates in ipRGCs was assumed, perhaps not unreasonably, to play a role in controlling the release of dopamine from these cells. However, research from our group has shown that light-dependent release of dopamine in the retina is neither driven by, nor dependent on, melanopsin phototransduction (Cameron et al., 2009) and we show that rod photoreceptor input is the major driver of light-dependent release (Perez-Fernandez et al., 2019). This was an unexpected finding and suggests that either release of dopamine in the retina is not linearly correlated with the spiking activity of DA cells or that the ipRGC input is confined to a subset of DA cells that do not release dopamine. Indeed, ipRGC input has only been found in ∼20% of tyrosine hydroxylase-containing cells (Zhang et al., 2008), which potentially have a different function, despite displaying no obvious differences in morphology. Similar to dopaminergic cells in the rest of the brain, DA amacrines also release GABA (Hirasawa et al., 2015) and have recently been shown to express vesicular nucleoside transporter (VNUT) that is responsible for vesicular ATP release (Ho et al., 2015). Therefore, the question remains as to the significance of this ipRGC intraretinal signaling and how it may influence retinal physiology through other neurotransmitter systems. Interestingly, TH has been shown to co-express with melanopsin in displaced ipRGCs of the cone-dominated tree shrew retina (Johnson et al., 2019) and while the significance of this is not yet known, it suggests an intimate relationship between these two systems. Indeed, while this does not occur in the mouse or rat retina, ipRGC dendrites form a plexus with TH cell processes in the OFF stratum of the IPL (Vugler et al., 2007).

## Gap junction connectivity

Another way ipRGCs have been shown to participate in intraretinal signaling is through gap junctions. Gap junctions are direct electrical synapses between cells made up of a pair of plasma membrane hemichannels (connexons) that directly connect the cytoplasm of neighboring cells allowing molecules and ions to flow between cells. Gap junction coupling between ipRGCs, and other inner retinal neurons, was originally shown in flat-mount mouse retinae lacking rods and cones (*rd/rd* cl) (Sekaran et al., 2003). Calcium imaging revealed that light caused an increase in intracellular calcium in ∼3% of cells in the ganglion cell layer, but when carbenoxolone, a gap junction blocker, was added the population of responding cells was reduced by 56%. They suggested that ipRGCs form an extensive network in the ganglion cell layer with other ganglion cells and/or displaced amacrine cells via gap junction coupling (Sekaran et al., 2003). Subsequently, questions over the selectivity of carbenoxolone have been raised (Bramley et al., 2011), however, whole-cell recordings of ipRGCs in mice treated with meclofenamic acid (MFA; thought to only block gap junctions) showed an increase in cell input resistance suggesting that ipRGCs possess electrical synapses with other cells (Schmidt et al., 2008). Additionally Eleftheriou et al. (2020) recorded light responses from almost 40% of RGCs using multi-electrode array (MEA) recordings in the *rd/rd Cnga1^–/–^* animals that lack rod and cone function; this was reduced to ∼10% with the addition of MFA suggesting widespread coupling of ipRGC to RGCs in these animals. Further, gap junction coupling between ipRGCs and displaced amacrine cells and possibly other ganglion cells in the ganglion cell layer was confirmed using neurobiotin tracer injections, specifically in the M1-M3 subtypes (Muller et al., 2010). Similar coupling was observed with neurobiotin injection in macaque and human ipRGCs confirming this phenomenon is not limited to rodent retinae (Liao et al., 2016).

IpRGCs in rats have been shown to provide input to spiking, sustained, ON displaced amacrine cells in the ganglion cell layer through gap junctions (Reifler et al., 2015). The responses recorded in the displaced amacrine cells were sustained, sluggish, had a spectral sensitivity at 480 nm, survived the pharmacological blockade of rod-cone signaling and were observed in degenerate retinas lacking rods and cones, confirming that they are driven by ipRGCs (Reifler et al., 2015). The addition of MFA abolished the ipRGC-driven inputs confirming they were gap junction mediated (Reifler et al., 2015). Sustained input to an analogous amacrine cell type in the mouse was also identified that co-stratifies neatly with M2 ipRGCs; this response persisted under chemical synaptic block suggesting these cells are driven via electrical synapses from ipRGCs (Sabbah et al., 2017).

A more recent study in mice has shown that all six subtypes of ipRGCs (M1-M6) exhibit tracer coupling with amacrine cells (ACs) but, specifically, none of the labeled cells were ganglion cells, as assessed by co-labeling with RGC marker, RNA-binding protein with multiple splicing (RBPMS) (Harrison et al., 2021). The labeled amacrines were of various soma diameters, suggesting a variety of coupled amacrine cell types. There was some correlation with ipRGC type e.g., M4s coupled with a higher proportion of large soma amacrines and ∼90% of the ipRGC coupled amacrine cells were found in the ganglion cell layer (the rest in the inner nuclear layer), however, displaced ipRGCs were not injected and so this percentage may be GCL skewed. Some of these coupled ACs have been shown to express neuropeptide Y, nitric oxide synthase or accumulate serotonin, suggesting that this intraretinal ipRGC to amacrine cell signaling could possibly exert a diverse modulatory effect on retinal physiology (Harrison et al., 2021). The release of these neuromodulators in response to ipRGC activation, however, remains to be investigated.

**FIGURE 2:**
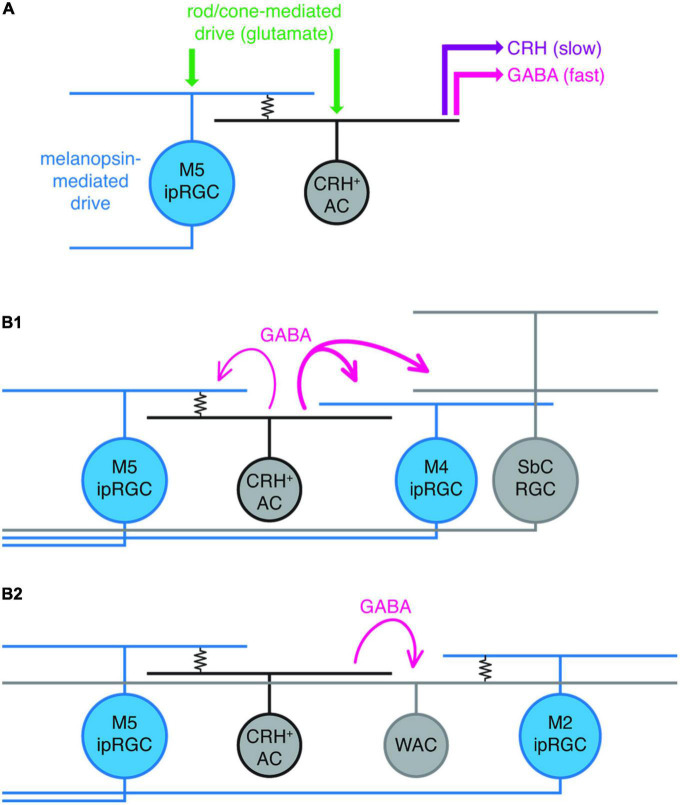
Summary of hypothesized ipRGC intraretinal connectivity of M5 ipRGCs in the mouse retina. **(A)** M5 to corticotropin-releasing hormone-expressing amacrine cell (CRH^+^ AC) electrical coupling (resistor symbol), in addition to glutamate-mediated excitatory input to both M5 ipRGCs and CRH ACs (originating from rods and cones), drives GABA and CRH release from CRH ACs. **(B1)** Strong (thick magenta lines) feedforward GABAergic inhibition of M4 ipRGCs and a suppressed-by-contrast (SbC) RGC from an M5 coupled CRH^+^ AC; modest (thin magenta) GABAergic feedback inhibition to an electrically coupled M5 ipRGC. **(B2)**, M5 ipRGC electrically coupled to a CRH + AC drives feedforward inhibition of a wide-field amacrine cell (WAC) that is electrically coupled to an M2 ipRGC. Reproduced with permission from Pottackal et al. (2021).

The most functional evidence so far for the role of ipRGC gap junction coupling comes from a recent paper from Pottackal et al. (2021) who describe electrical synapses between M5 ipRGCs and corticotropin-releasing hormone-expressing (CRH^+^) amacrine cells. Interestingly, CRH^+^ ACs release GABA upon receiving light-mediated excitatory inputs from M5 ipRGCs which provides local, and wide-field lateral suppression of the retinal output either *via* (i) GABAergic feedback inhibition (from CRH^+^ ACs back to M5 ipRGCs), (ii) feedforward direct GABAergic inhibition of M4 ipRGCs and suppressed-by-contrast (SbC) RGCs (Park et al., 2018), or (iii) feedforward indirect inhibition of M2 ipRGCs and non-ipRGCs via CRH^+^ AC GABAergic inhibition of intermediate wide-field ACs (Figure 2). This study strongly indicates that ipRGCs, via electrical synapses with interneurons, influence the retinal circuitry in a complex and wide-ranging fashion (Pottackal et al., 2021). This work specifically concentrated on the feedback/feedforward influence of just one type of coupled amacrine cell (CRH^+^ AC), but it is likely the other ipRGC gap junction connections discussed above also influence retinal circuitry in similarly complex ways involving multiple excitatory and inhibitory loops.

In the developing retina prior to eye opening many subtypes of ipRGC are extensively coupled to other ipRGCs and non-ipRGCs. These gap junctions transmit slow photocurrents and spikelets of the intrinsic light response between ipRGCs and are essential for the generation of full-amplitude calcium responses in the majority of functional neonatal ipRGC classes (Caval-Holme et al., 2019). Interestingly, M1 ipRGCs exhibited little or no tracer coupling in neonates and blockade of gap junctions with MFA did not change their light response. Heterotypic ipRGC and non-ipRGC coupling between the other morphological types, however, was extensive. This gap junction connectivity was increased by the dopamine antagonist, suggesting a role for retinal waves in modulating the sensitivity of ipRGCs by driving dopamine release (Arroyo et al., 2016). While it is not immediately apparent why an increase in gap junction connectivity of cells with small intrinsic light responses (when gap junctions are closed) generates higher amplitude calcium and spiking responses, it is possible that non-linear synergistic interactions between voltage-dependent conductances and gap junction conductances enhances the light responses (Caval-Holme et al., 2019).

## ipRGC intraretinal/interocular signaling in development

Mammals express melanopsin long before rod and cone pigments are detectable, making melanopsin photosensitivity the earliest means of phototransduction recorded in the retina (Tarttelin et al., 2003). Rods and cones become photosensitive around postnatal day 10 (P10), whereas ipRGCs are photosensitive before birth (Rao et al., 2013). Early postnatal ipRGCs show Ca^2+^ signals in response to light, and neonatal ipRGCs appear to be functionally connected to the SCN (Weaver and Reppert, 1995; Hannibal and Fahrenkrug, 2004; Sekaran et al., 2005; Lupi et al., 2006; Caval-Holme et al., 2019). Melanopsin photosensitivity has been shown to regulate the development of retinal neurons, retinal vasculature and retinogeniculate circuits including negative phototaxis which causes mouse pups (as early as P6) to turn away from bright light (Johnson et al., 2010).

There are far more ipRGCs at birth than in the adult retina, with a neonatal density of ∼200/mm^2^ dropping by 70% to an adult density of ∼60/mm^2^ by P14 (Sekaran et al., 2005; Gonzalez-Menendez et al., 2010). When mouse pups are deprived of light or melanopsin (either dark-reared or *Opn4*^–/–^), the number of ganglion cells is increased when the animals reach adulthood suggesting a deficit in developmentally appropriate neuronal loss. Further, this lack of input from ipRGCs in development results in delayed regression of the embryonic hyaloid vasculature and overgrowth in the adult retinal vasculature (Rao et al., 2013). The mechanism linking these vascular changes to ipRGC activation is unclear but is thought to involve modulation of vascular endothelial growth factor (VEGFA), a potent signal for vascular endothelial survival. Perhaps the most surprising result from this study is that the most critical time for ipRGC activation to drive normal development is at, or before, embryonic day 16-17 (E16-17), when the pup is still *in utero* (4-5 days before birth). This effect was specific to light activation of these embryonic ipRGCs, and not those of the mother, meaning light levels *in utero* are sufficient to activate ipRGCs, and is necessary for normal development.

Immature melanopsin-expressing retinal ganglion cells extend their dendrites to the OPL in developing retina (Renna et al., 2015). These outer retinal dendrites (ORD) originate exclusively from conventional M1 ipRGCs in the ganglion cell layer and displaced M1 ipRGCs in the inner nuclear layer, can be branched or unbranched, and are morphologically distinct (Sondereker et al., 2017). ORDs can be observed as early as P4, with peak incidence between P8 and P12. Densities decrease in adulthood; however, some still remain (Renna et al., 2015; Sondereker et al., 2017). M1 and displaced M1 ipRGCs and their ORDs are distributed asymmetrically with their peak density in the dorsal retina (Hughes et al., 2013). Dendrites of the ORDs lie in close apposition with cone photoreceptor terminals in the OPL, and express post-synaptic glutamate receptors and postsynaptic density protein 95 (PSD-95) suggesting a direct synaptic connection of ipRGCs with cones (Sondereker et al., 2017). The significance of the prevalence of these connections in the dorsal retina is not known but may be related to the asymmetry of M- and S-opsin (Applebury et al., 2000).

Indeed, light activation of ipRGCs in early development has been shown to control correct cone lamination in the developing mouse retina (Tufford et al., 2018). Ablation of ipRGCs during development using Diphtheria toxin-A in *Opn4^DTA/DTA^* mice causes S- opsin expressing cones and some M-cones to be displaced, and their expression is detected outside the outer nuclear layer. The positioning of cones within the ONL was also affected and ipRGC ablation caused an increase in the expression of cone arrestin and hence a downregulation of cone photosensitivity. This suggests that light-evoked ipRGC responses play a major role in neuronal lamination in postnatal week 1 before rods and cones develop their photosensitivity (Tufford et al., 2018). There is some indication that retinal dopamine levels play a role, with altered dopamine levels in *Opn4^DTA/DTA^* mice due to a lack of input to DA amacrines. While development of dopaminergic amacrine cells is not affected by the loss of melanopsin or ipRGCs (Munteanu et al., 2018), dopamine release in animals lacking ipRGCs has not been investigated. However, adult *Opn4^DTA/DTA^* animals lack adaptation of the cone ERG which is thought to be driven by dopamine release (Prigge et al., 2016).

IpRGCs also play a role in spontaneous waves of retinal activity that propagate across RGCs in early postnatal stages contributing to the refinement of connectivity between the retina and dLGN. Melanopsin activation extends these waves under normal lighting conditions (Renna et al., 2011; Kirkby and Feller, 2013) and their ablation impairs connectivity of the retina to the brain reducing visual acuity (in adulthood) and prevents light from setting the endogenous period of the circadian clock during development (Chew et al., 2017).

Taken together, this research underscores the importance of understanding the influence of ipRGC intraretinal, and intraocular signaling, in development. Importantly, how does modern artificial lighting conditions and an indoor lifestyle affect embryonic/neonatal ipRGC activation, and subsequently the neural development of vision?

## ipRGCs and myopia

Research into retinal outputs that influence myopia development or progression has a long and complex history. It is known that changes in the refractive index of the eye can be driven without the influence of the rest of the nervous system (Troilo et al., 1987; Norton et al., 1994; Mcfadden and Wildsoet, 2020) and that photoreceptor activity is implicated, at least in part, in driving these adaptive responses. Little research has focused specifically on the importance of ipRGC activation to refractive development and it has mainly been conducted in mice. It is clear that ipRGC function is of importance to both natural myopic shifts occurring in development, and the induction of myopia via form-deprivation (FDM) where the animal has de-focus imposed via a diffusing lens applied to the eye (Chakraborty et al., 2022; Liu et al., 2022). Refractive development can be altered by the light environment even in the absence of rods and cones (Liu et al., 2022), suggesting ipRGCs are communicating this information. However, removing ipRGCs from the equation has produced conflicting results depending on the way in which ipRGC function was eliminated, the controls used to compare myopic/hyperopic progression, the developmental time point assessed, and the methodologies used to induce FDM. Thus, so far, no clear conclusions can be drawn about the precise role of ipRGC activation in refractive development and this is likely due to the complex push-pull nature of myopia generation that is still mechanistically not well understood. Lui et al. saw that ablation of ipRGCs caused a myopic shift and, conversely, chemogenetic activation of ipRGCs a hyperopic shift (Liu et al., 2022). Chakraborty *et al*., however, see hyperopic shifts in both *Opn4^–/–^* and *Opn4^DTA/DTA^* animals. These differences in results are perhaps not surprising given the role of ipRGCs in retinal development, and the way in which ipRGC function has been eliminated in these models: *Opn4^DTA/DTA^* animals are lacking ipRGC neurons from, or before, birth; *Opn4^–/–^* animals retain ipRGC cells but experience the developmental changes discussed above; and the ablation of ipRGCs by Lui et al. occurred at P18 and was not complete. However, it is clear that ipRGC intraretinal, and intraocular signaling plays a key role in refractive development. Certainly, a role for short wavelength light in preventing myopia has been identified in many animals including mammals (Long et al., 2009; Foulds et al., 2013; Qian et al., 2013; Jiang et al., 2014), however, other novel photopigments such as Opn3 and Opn5 are also blue/UV sensitive, and Opn5 has recently been shown to play a significant role in refractive development (Jiang et al., 2021), making interpretation of this result more complex.

## Effect of ipRGC activation on the electroretinogram

The electroretinogram (ERG) is a non-invasive tool that can be used to measure the electrical response of rod and cone early pathways in the retina. Before the photopigment driving the photoresponse in ipRGCs was generally accepted as melanopsin, Hankins and Lucas described the effect of blue light on the temporal properties of cone photoreceptors using the ERG. They showed that the cone ERG had a delayed b-wave peak when recorded at midnight as opposed to midday, and that this circadian rhythm could be attenuated by prior light exposure in the subjective night (15 mins). This effect had a high threshold and a spectral sensitivity at 480 nm, suggesting that it was ipRGC stimulation that was modulating cone signal processing in the retina. Interestingly, a smaller, but significant, reduction in b-wave implicit time was observed in the contralateral eye, despite occlusion of that eye from the adapting stimulus, suggesting that ipRGC influence may extend to the contralateral eye (Hankins and Lucas, 2002).

Subsequently, ERG recordings of the cone pathway in both wild-type and melanopsin knockout mice showed significant differences in both amplitude and speed of the b-wave between midday and midnight, suggesting that these modifications are determined by both long-term light history and the circadian clock. Converse to the human results, however, melanopsin knockout mice showed a lack of circadian rhythmicity of the cone ERG but retained modulation by light history (Barnard et al., 2006) suggesting that melanopsin/ipRGCs play a role either in maintaining circadian rhythmicity of the retina or the influence of ipRGCs on cone pathways is gated by a retinal circadian clock.

Since deletion of melanopsin causes the widespread changes in retinal development discussed above, Allen *et al*. further characterized the influence of ipRGC activation on the mouse ERG using an animal with an intact retinal system (Allen et al., 2014). They used the approach of receptor silent-substitution, which exploits the differences in spectral power distributions of individual photopigments (Rushton, 1972; Estévez and Spekreijse, 1982). Thus, using polychromatic stimuli, they were able to present pairs of equivalent cone stimuli (matched in irradiance and contrast) that differed in their activation of melanopsin. Using these stimuli, they found that the cone ERG b-wave amplitude was inversely correlated with melanopsin activity, with significantly lower cone ERG b-wave amplitudes under high melanopsin activation, suggesting a suppressive effect of ipRGC activation on cone ERG output. In turn, they saw that these effects were relayed to the visual thalamus, whereby changes in ipRGC activity adjusted feature selectivity in visual circuits in both spatial and temporal dimensions (Allen et al., 2014).

More recently, targeted gene delivery of chemogenetic Gq-coupled receptor, hM3Dq, to ipRGCs in *Opn4^Cre/+^* mice allowed specific activation of ipRGCs in the dark using intraperitoneal injection of Clozapine-N-oxide (CNO). ipRGC activation suppressed both the a- and b-wave amplitudes of the dark-adapted ERG indicating that the influence of ipRGCs on retinal physiology may extend to the photoreceptors as well as their downstream pathways (Milosavljevic et al., 2016). While ERGs are measured *in vivo* and the effects of ipRGC activation may result from retinopetal innervation from higher brain areas (Gastinger et al., 2006; Akimov et al., 2010) the stimulus originates in ipRGC photoreceptors.

## Influence of melanopsin on ganglion cell output

While M1 and M2 ipRGCs have clear non-image-forming functions (circadian rhythm entrainment, pupil light response) M4-M6 ganglion cells participate in image-forming vision tasks including contrast detection and color-opponency. How might melanopsin contribute to these functions given that it is co-expressed in these cells? The intracellular influence of melanopsin signaling within ipRGCs has been further investigated in the M4 subtype (sustained ON-alpha RGC), which act as a contrast detector (Schmidt et al., 2014; Sonoda et al., 2018). Intrinsic melanopsin responses in M4 ipRGCs, although very small and sluggish (Ecker et al., 2010; Estevez et al., 2012; Schmidt et al., 2014), allow these cells to signal ambient light intensities over long periods of time (Brown et al., 2010; Schmidt et al., 2014). Schmidt et al. showed that this property optimizes classical photoreceptor-driven contrast sensitivity as mice lacking melanopsin exhibited deficits in contrast sensitivity both at a behavioral level and when measuring from the M4 cells themselves (Schmidt et al., 2014; Sonoda et al., 2018). Furthermore, chemogenetic activation of Gq pathways (the pathway which is endogenously activated by melanopsin) in *Opn4^Cre/Cre^* mice rescued the contrast sensitivity of M4 ipRGCs to wild-type levels confirming that the contrast sensitivity deficits are not a consequence of developmental circuit rewiring. The mechanism driving melanopsin’s optimization of M4 function was found to be an increase in input resistance of M4 cells resulting from a G-protein meditated closure of potassium leak channels (suggested to be TASK K2P). This occurred under dim light conditions and scaled with background light intensity. This increase in input resistance, while not causing an obvious measurable change in membrane potential, enhances the impact of synaptic inputs on the membrane potential, essentially augmenting rod and cone originating signals to optimize contrast detection at different ambient light conditions. Under brighter light conditions, melanopsin triggers the opening of TRP channels that boosts depolarization of the M4 cell in response to light, further enhancing contrast detection. In this sense, melanopsin is acting like a photographer’s light meter to determine the best operating conditions to optimally detect contrast in these specialized alpha-RGCs.

Interestingly, the authors found that melanopsin does the opposite in M1 ipRGCs under dim light and that activation actually reduces their intrinsic excitability by opening TRP channels and decreasing the input resistance. In contrast to M4 cells, they find no evidence that potassium leak channels are closed in M1 cells. This mechanism may therefore limit the impact of rod and cone responses on the output of M1 cells under dim ambient light conditions, potentially gating their signals to high thresholds and relying predominately on melanopsin signaling. This would suit the role of M1 cells in circadian entrainment as the melanopsin “photon counting” signal would be more reliable to the clock than the rod and cone response that adapts to ambient light conditions. However, it is clear that rods and cones have an important role in both circadian entrainment and the pupil light response (Lall et al., 2010; Keenan et al., 2016).

These intrinsic cellular mechanisms may have important consequences for the final output of ipRGCs over the different ambient light conditions experienced by the retina over the course of the day. Milosavljevic *et al*. showed that ipRGCs adjust their intrinsic firing rate based on irradiance and influence the firing rate of ∼30% of other RGCs. This may be a mechanism to control the information transfer capacity of the optic nerve by increasing or decreasing visual gain based on irradiance (Milosavljevic et al., 2018). When no other stimulus is present, an increase in ambient light intensities increases the basal firing rate of RGCs and this is disrupted in *Opn4^–/–^* mice lacking melanopsin (Milosavljevic et al., 2018). As spike firing and associated synaptic activity accounts for 80% of energy expenditure in the brain (Attwell and Laughlin, 2001; Lennie, 2003), irradiance-dependent modulation of RGCs appears to be a mechanism for trading off information content with energetic cost. Thus, ipRGC signaling is involved in controlling the rate of information flow in the optic nerve (Milosavljevic et al., 2018).

## Conclusion

**FIGURE 3:**
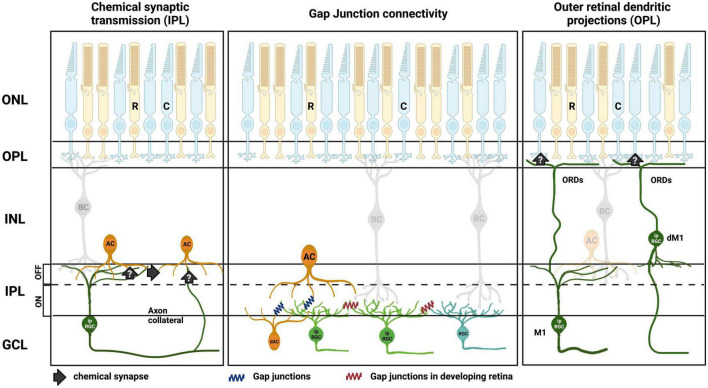
Summary of ipRGC intraretinal signaling routes in the retina. **(Left)** Chemical synaptic transmission to amacrine cells (AC) is either direct, via axon collaterals (Joo et al., 2013), or indirect via intermediary amacrines. **(Middle)** Direct gap junction connection of ipRGCs to amacrine cells in the GCL and INL in adult retina. Direct gap junction connection of ipRGC to amacrine cells (Muller et al., 2010; Reifler et al., 2015; Harrison et al., 2021; Pottackal et al., 2021), and ipRGC to ipRGCs and non-ipRGCs, during retinal development (Arroyo et al., 2016; Caval-Holme et al., 2019). **(Right)** Putative synaptic inputs to cone photoreceptor terminals in the OPL via outer retinal dendrites (ORDs) from M1 ipRGCs (Renna et al., 2015; Sondereker et al., 2017). Outer nuclear layer (ONL), outer plexiform layer (OPL), inner nuclear layer (INL), inner plexiform layer (IPL), ganglion cell layer (GCL), rods (R), cones (C), bipolar cell (BC), displaced amacrine cell (dAC), displaced M1 ipRGC (dM1). Created with BioRender.com.

Since their discovery over 20 years ago, it has become clear that, alongside their well-established role as retinal output neurons, ipRGCs play a parallel role within the retina where they adjust the behavior of other retinal neurons. As highlighted in this review, we now know that ipRGCs make both chemical synaptic and gap junction connections to communicate with neurons throughout the retinal network as summarized in Figure 3. This has the potential to drive widespread changes in the cells and circuits that support non-image-forming and image-forming vision alike, plus a clear role in the development of the visual system. Since melanopsin allows ipRGCs to better encode ambient light levels than rods and cones, which more readily adapt and discard this information, a potential role for ipRGCs controlling adaptational mechanisms in the retinal network is clear. The influence of ipRGCs on the development of the retina and other ocular structures, meanwhile, suggests a vital role of ipRGCs in more gross features of the visual system.

Understanding the mechanisms and routes by which ipRGCs communicate throughout the retina and other ocular structures will give us a more complete understanding of vision, from development through to functional outputs. Importantly, though, this research allows greater appreciation of the implications of lighting design on these critical aspects of vision. Indoor lighting can vary substantially in its spectral composition and intensity, which often results in reduced activity of melanopsin (and therefore ipRGCs) compared to natural daylight (Brown et al., 2022). We do not have a clear view of how this might alter the normal function of the retina, but the evidence presented in this review suggests that there could be widespread changes in retinal functioning when ipRGC activity is altered, particularly in development. Future work will reveal implications for such outputs, and in turn, be used to improve the design and performance of artificial lighting and visual displays for the benefit of vision.

## Author contributions

SR and MC wrote the manuscript and compiled Figure 1. SR and NM complied Figure 3. AA and NM edited and contributed to the manuscript. All authors contributed to the article and approved the submitted version.
